# Supplementary material for: Coevolution of Cooperation and Partner Rewiring Range in Spatial Social Networks
Source: Sci Rep. 2016 Nov 8;6:36293. doi: 10.1038/srep36293 (PMC5099781; doi:10.1038/srep36293)
Supplement: Supplementary Information [file srep36293-s1.pdf]

Coevolution of Cooperation and Partner Rewiring Range in Spatial Social Networks

Tommy Khoo, Feng Fu, Scott Pauls

Supplementary Information

List of Figures

1 Degree heterogeneity over time. 2
2 Comparison of average node distance. 3
3 Comparison of average clustering coefficient. 4
4 Strategy and density of highest degree nodes. 5
5 Optimal availability of reputation information for defectors. 6
6 Global vs local cooperators at high values of p. 7
7 Non-binary rewiring range preference. 8
8 Global rewiring, local rewiring and both under coevolution, under different costs. 9
9 Time evolution of proportion of GC, LC, GD, and LD under $c = 0.1$. 10
10 Time evolution of proportion of GC, LC, GD, and LD under $c = 0.5$. 11
11 Change in average path length as number of nodes increases. 12
12 Bilateral link creation version of Figure 2. 13
13 Bilateral link creation version of Figure 3. 14
14 Bilateral link creation version of Figure 4. 15

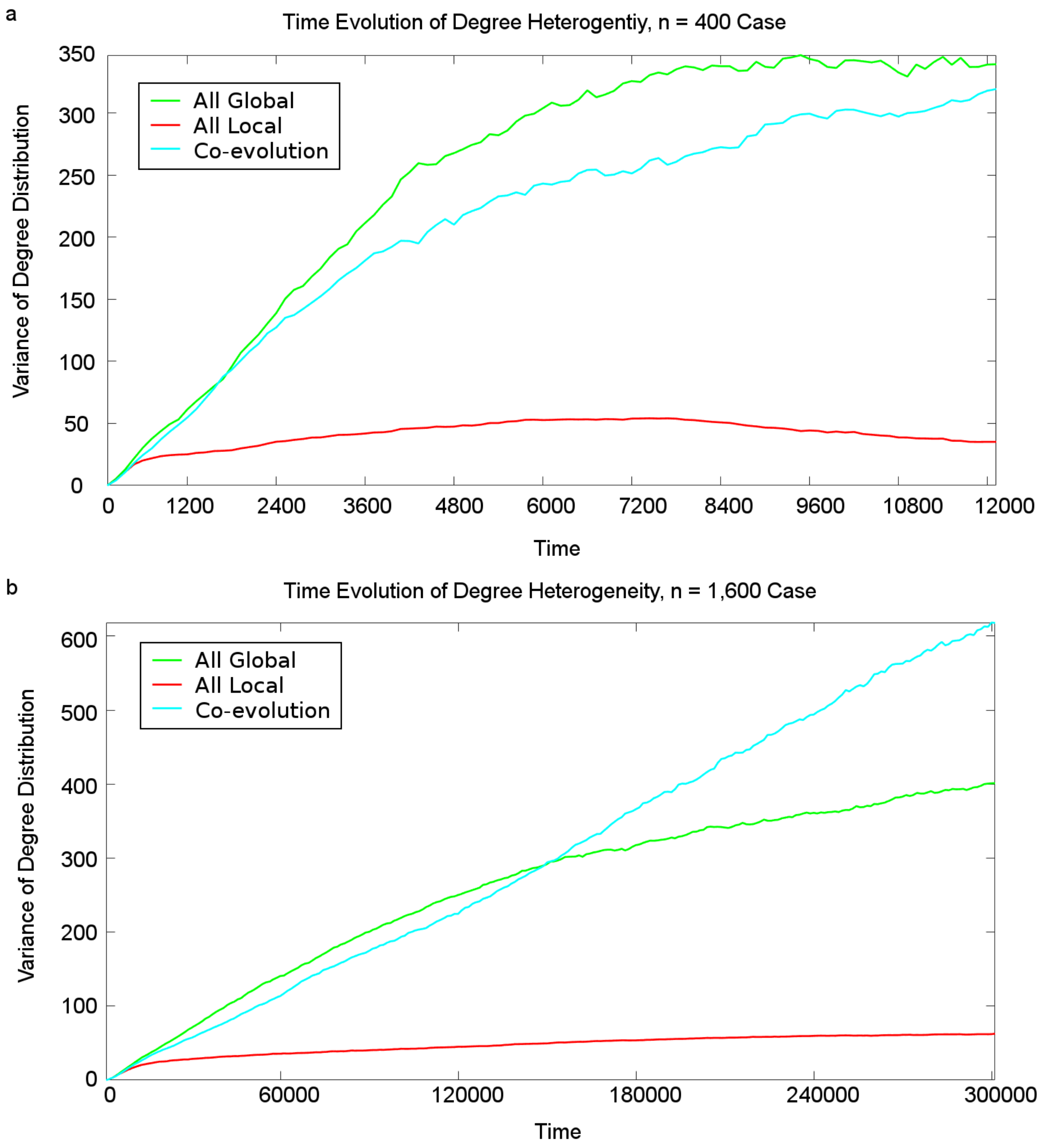

Supplementary Figure 1. **Degree heterogeneity over time.** Figures show the variance of the degree distribution over time for only global rewiring, only local rewiring, and both under coevolution. In both Sup. Fig. 1 a and b, networks produced by only global rewiring and coevolution have degree distributions that increase in variance, and hence degree heterogeneity, over time. In contrast, networks produced by only local rewiring do not achieve the same level of variance. The network produced by coevolution in Sup. Fig. 1 b has a variance that eventually outgrow that of the network produced by only global rewiring. Simulation data in Sup. Fig. 1a was also used for Figure 2 in the main text. Parameters: $u = 0.1$, $w = 0.5$, $\beta = 0.1$, $p = 0.1$, $c = 0.2$, $d = 2$, $l = 1$, single run.

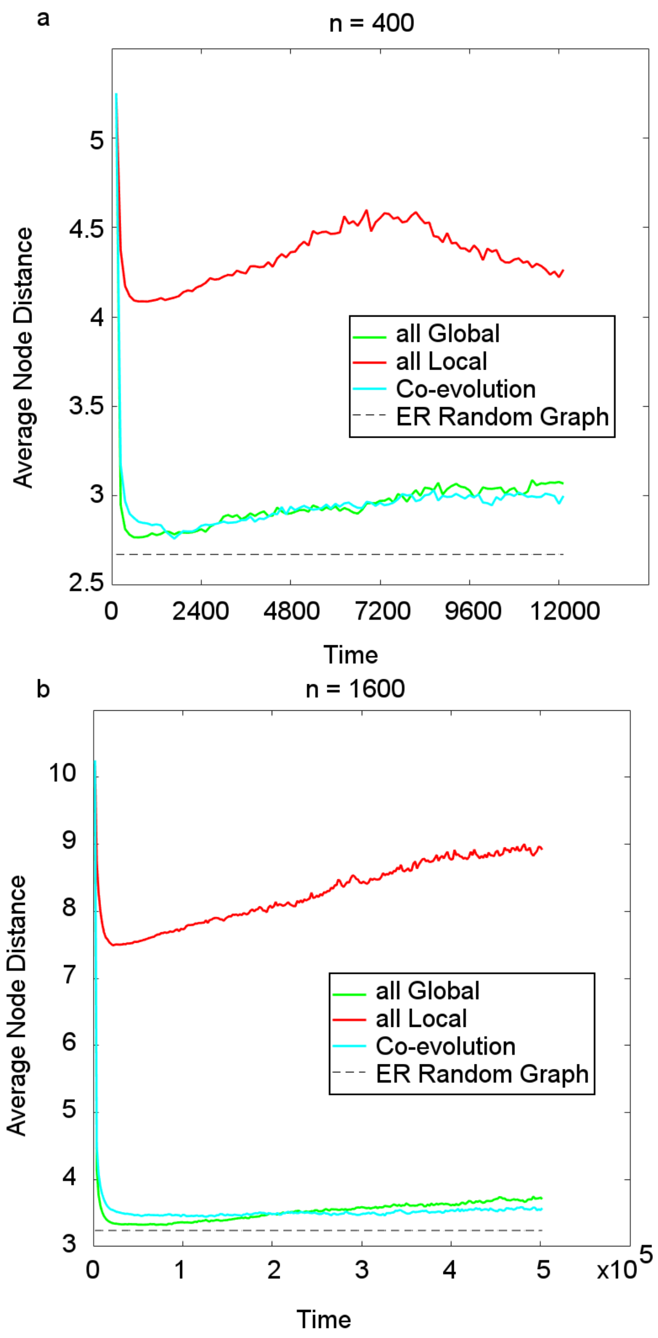

Supplementary Figure 2. **Comparison of average node distance.** Figures show the time evolution of average node distance (average of the shortest distance between nodes) for networks produced by only global rewiring, only local rewiring, and both under coevolution. In both Sup. Fig. 2 a and b, networks produced by only global rewiring and coevolution have average node distance close to that of an Erdős-Rényi (ER) random graph ensemble with the same average degree. On the other hand, average node distance remains much higher with only local rewiring. This is difference is more pronounced as the network size is increased from Sup. Fig. 2a $n = 400$ to Sup. Fig. 2b $n = 1600$. Simulation data in Sup. Fig. 2a was also used for Figure 2 in the main text. Parameters: $u = 0.1$, $w = 0.5$, $\beta = 0.1$, $p = 0.1$, $c = 0.2$, $d = 2$, $l = 1$, single run.

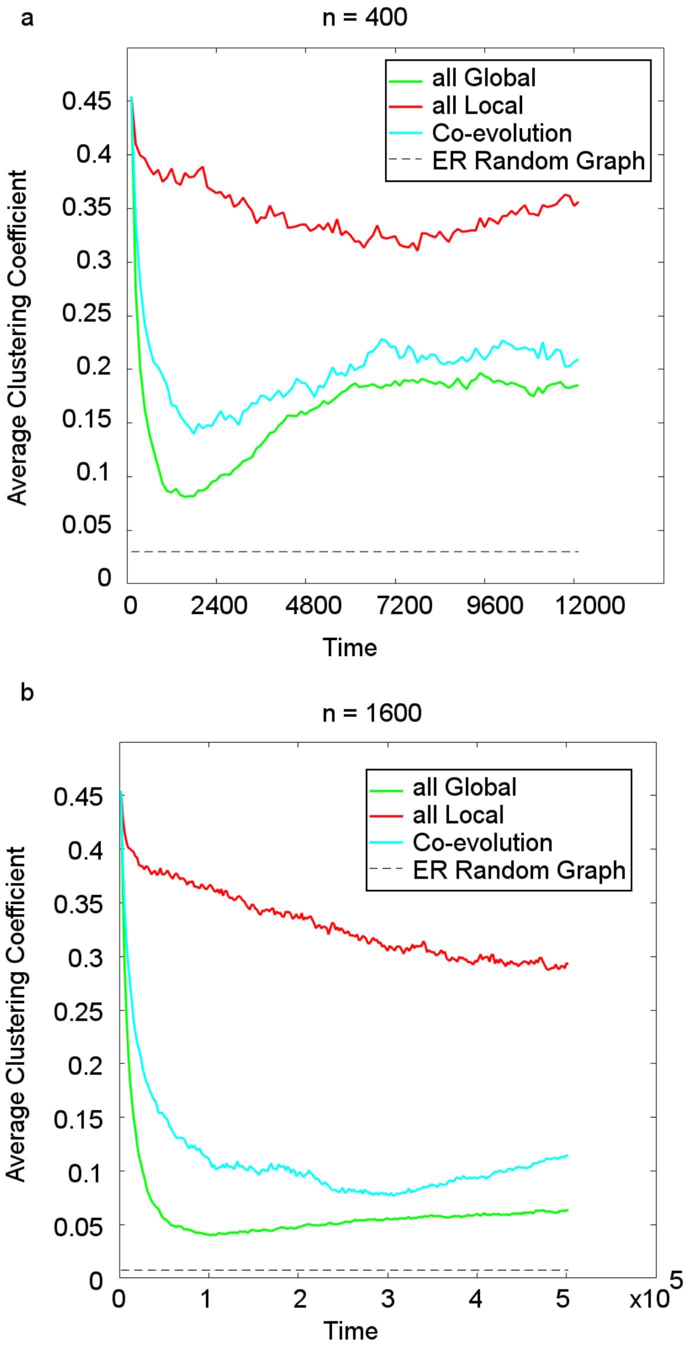

Supplementary Figure 3. **Comparison of average clustering coefficient.** Figures show the average clustering coefficient for networks produced by only global rewiring, only local rewiring, and both under coevolution. In Sup. Fig. 3b, networks produced by only global rewiring and coevolution have average clustering coefficient at least one order of magnitude greater than that of an Erdős-Rényi (ER) random graph ensemble with the same average degree. In Sup. Fig. 3a, these statistics are close to one order of magnitude greater. With only local rewiring, the clustering coefficient remains many orders of magnitude greater than the ER random graph ensemble in both Sup. Fig. 3a and Sup. Fig. 3b. Simulation data in Sup. Fig. 3a was also used for Figure 2 in the main text. Parameters: $u = 0.1$, $w = 0.5$, $\beta = 0.1$, $p = 0.1$, $c = 0.2$, $d = 2$, $l = 1$, single run.

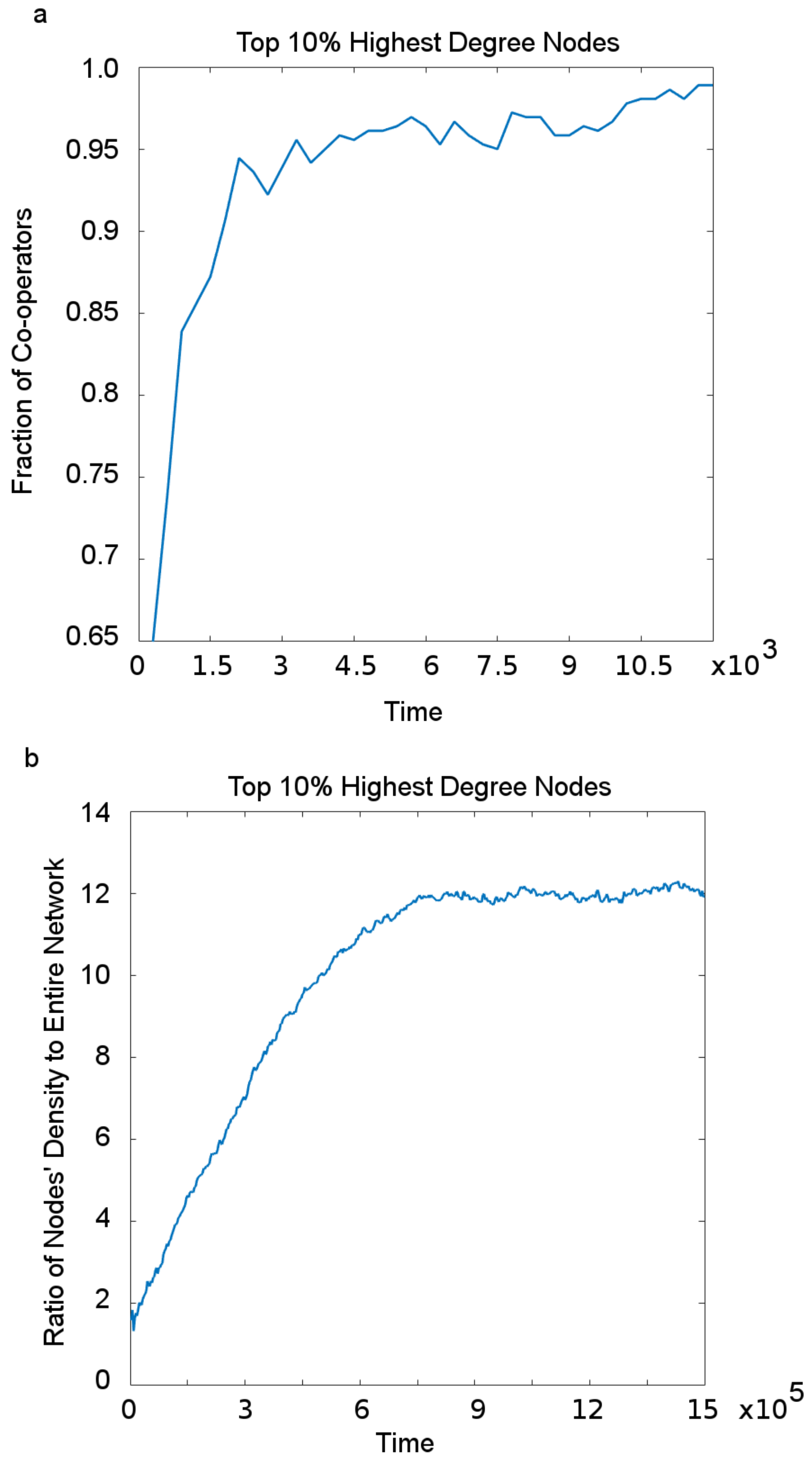

Supplementary Figure 4. **Strategy and density of highest degree nodes.** Sup. Fig. 4a shows the top 10% highest degree nodes becoming all cooperators early in the simulation. Sup. Fig. 4a is truncated at $t = 12 \times 10^3$ as the fraction of cooperators remained close to 1 for the rest of the simulation. Sup. Fig. 4b shows the ratio of the edge density (number of edges divided by number of possible edges) of the top 10% highest degree nodes to that of the entire network. Sup. Fig. 4b is truncated at $t = 15 \times 10^5$ as the ratio remained close to 12 for the rest of the simulation. Parameters: $n = 3600$, $t = 2 \times 10^6$, $u = 0.2$, $w = 0.2$, $\beta = 0.1$, $p = 0.15$, $c = 0.2$, $d = 2$, $l = 1$, single run.

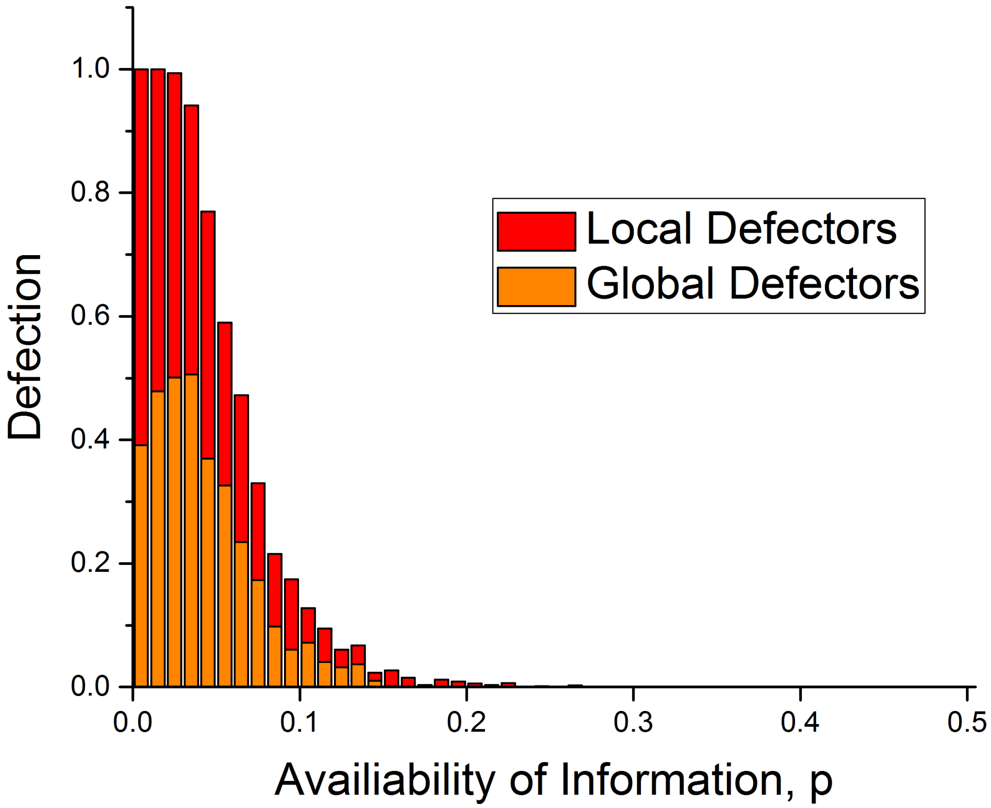

Supplementary Figure 5. **Optimal availability of reputation information for the evolution of global defectors.** A situation similar to Figure 4 happens for defectors. The proportion of global defectors increases with p, and reaches a global maximum, before falling again. Simulation data was also used for Figure 4 in the main text. Parameters: $n = 3600$, $t = 2 \times 10^6$, $u = 0.2$, $w = 0.2$, $\beta = 0.1$, $c = 0.2$, $d = 2$, $l = 1$, final result averaged over an additional 3×10^4 time steps, this process is then repeated for 100 runs.

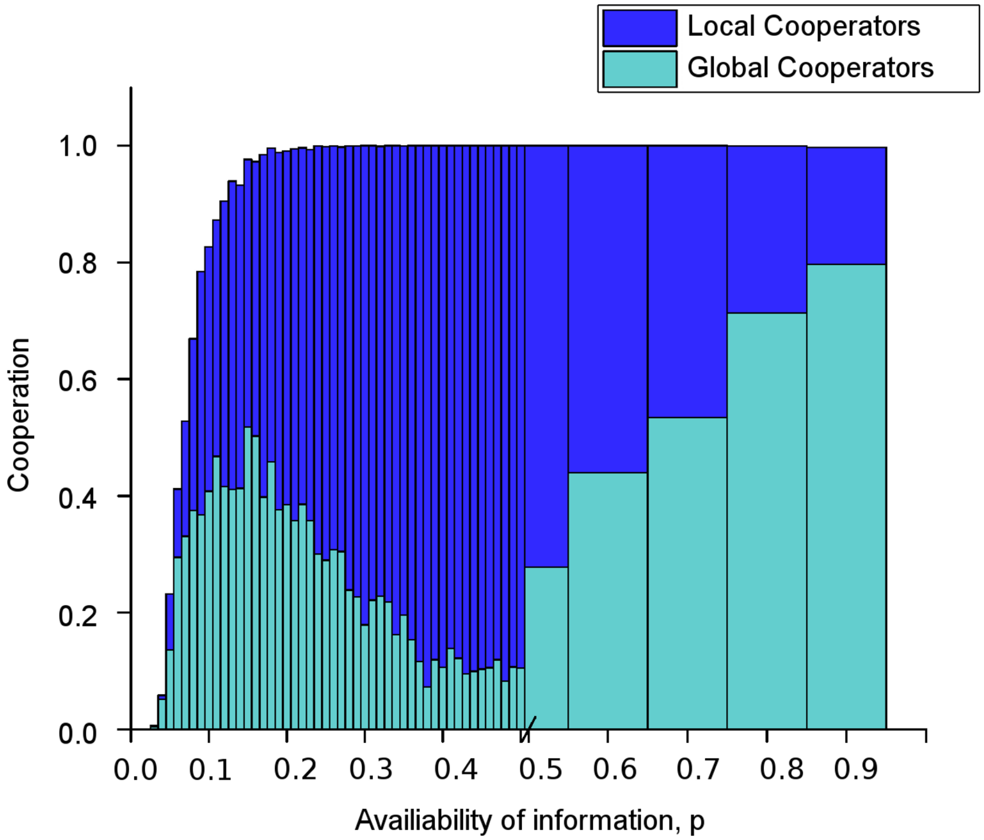

Supplementary Figure 6. **Global vs local cooperators at high values of** p**.** As parameter p is increased beyond $p = 0.5$, proportion of global cooperators increases monotonically. Simulation data was also used for Figure 4 in the main text. Parameters: $n = 3600$, $t = 2 \times 10^6$, $u = 0.2$, $w = 0.2$, $\beta = 0.1$, $c = 0.2$, $d = 2$, $l = 1$, final result averaged over an additional 3×10^4 time steps, this process is then repeated for 100 runs.

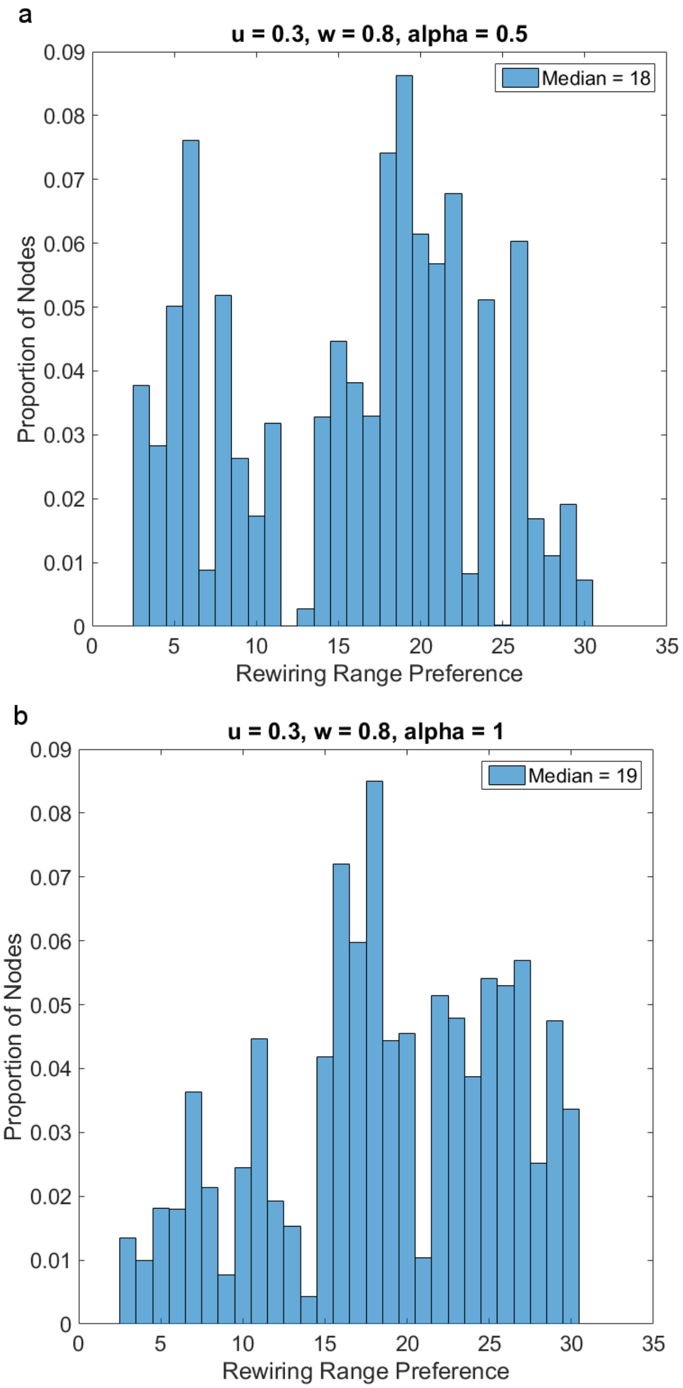

Supplementary Figure 7. Non-binary rewiring range preference. In Sup. Fig. 7 a and b, rewiring range preference is allowed to take values in $[d+1, n]$, initially assigned uniformly at random. The cost of rewiring is instead $(\frac{r_i}{n})^{\alpha}$, where r_i is the rewiring range preference of individual i, and Sup. Fig. 7a $\alpha = 0.5$ and Sup. Fig. 7b $\alpha = 1$. In both cases, the distribution of equilibrium rewiring range preference have a median, 18 and 19, that is higher than the mean and median, 16, of the uniform distribution on $\{3, 4, \ldots, 29, 30\}$. Parameters: $n = 900$, $t = 2 \times 10^6$, $u = 0.3$, $w = 0.8$, $\beta = 0.1$, $p = 0.1$, $c = 0.2$, $d = 2$, $l = 1$, combined over 100 runs.

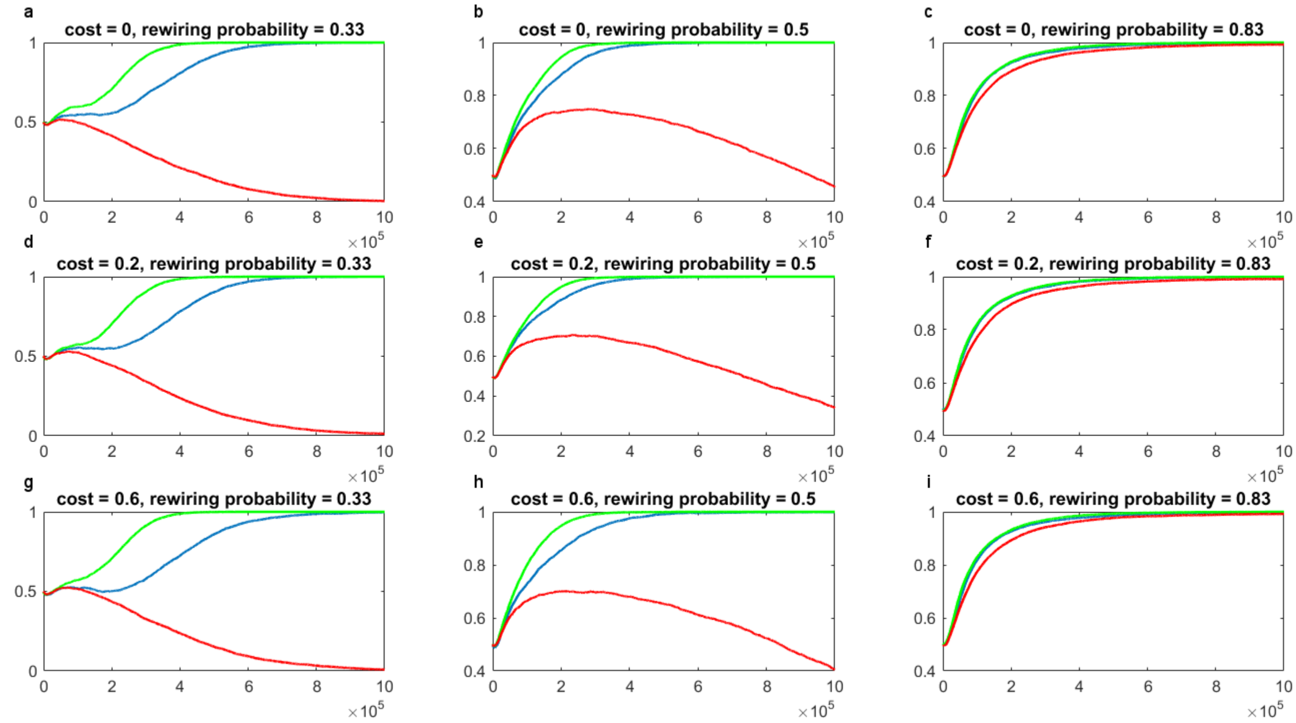

Supplementary Figure 8. **Global rewiring (green), local rewiring (red) and both under coevolution (blue), with different costs** c**.** Parameters: $n = 3600$, $t = 1 \times 10^6$, $u = 0.1$, $\beta = 0.1$, $p = 0.1$, $d = 2$, $l = 1$, averaged over 20 runs, lattice with non-periodic boundaries.

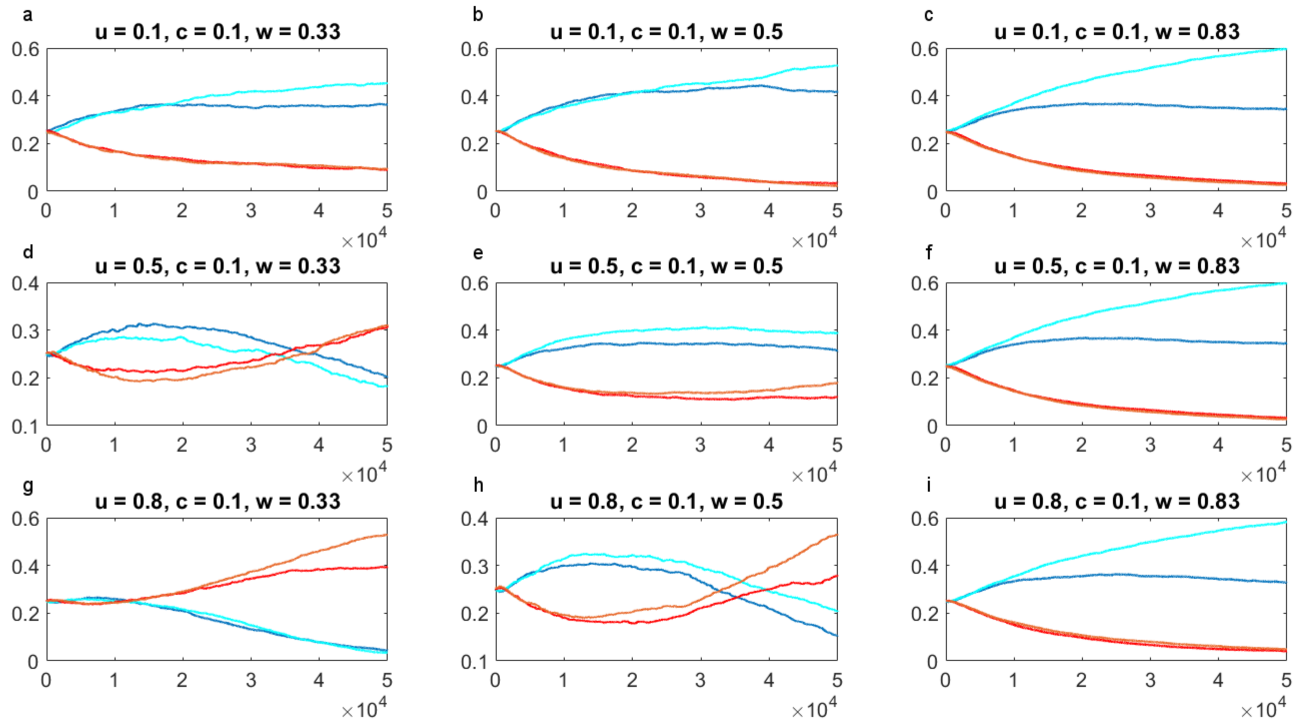

Supplementary Figure 9. **Time evolution of proportion of GC, LC, GD, and LD under** $c = 0.1$**.** Parameters: $n = 400$, $t = 5 \times 10^4$, $\beta = 0.01$, $p = 0.1$, $c = 0.1$, $d = 2$, $l = 1$, averaged over 100 runs, lattice with non-periodic boundaries.

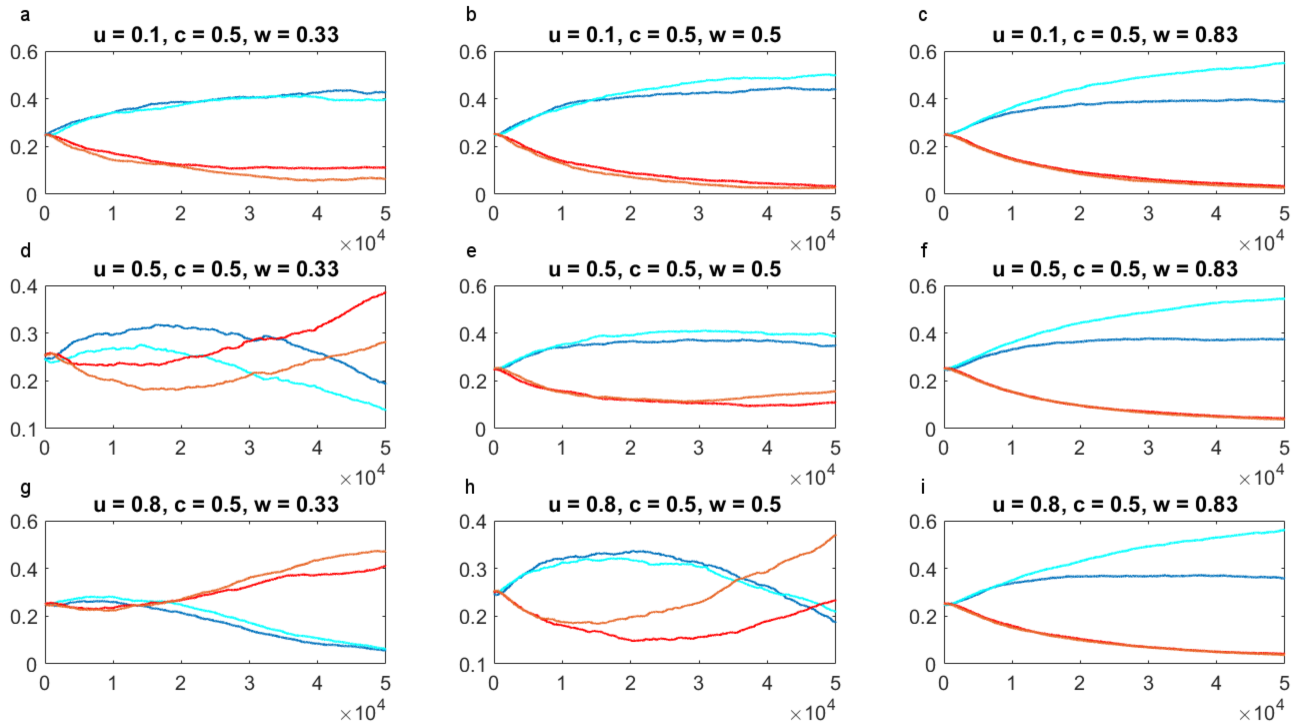

Supplementary Figure 10. **Time evolution of proportion of GC, LC, GD, and LD under** $c = 0.5$**.** Parameters: $n = 400$, $t = 5 \times 10^4$, $\beta = 0.01$, $p = 0.1$, $c = 0.5$, $d = 2$, $l = 1$, averaged over 100 runs, lattice with non-periodic boundaries.

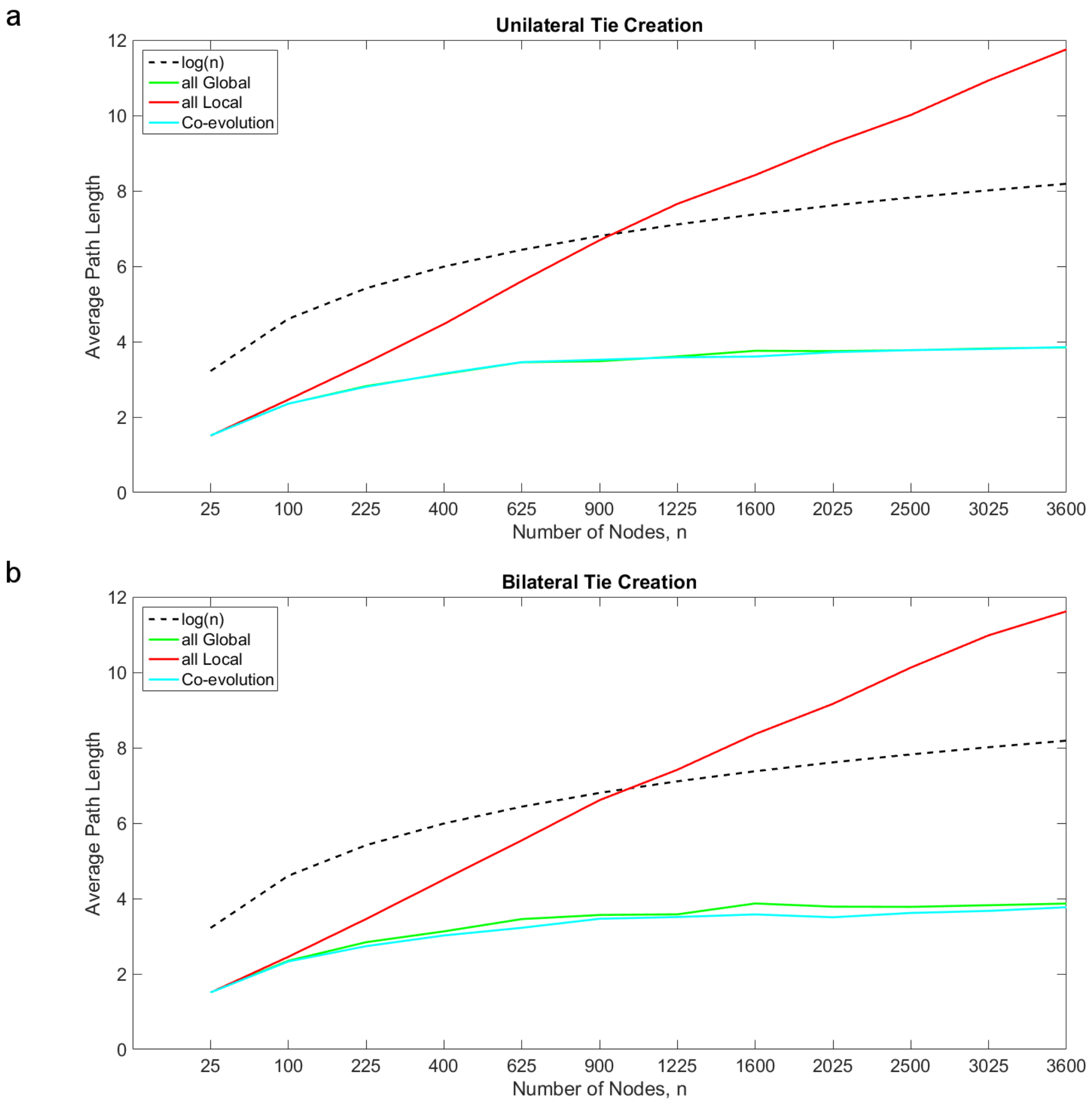

Supplementary Figure 11. **Change in average path length as number of nodes increases.** Figures show the average path length of the network at equilibrium as the number of nodes n increases, for only global rewiring, only local rewiring, and both under coevolution. Sup. Fig. 11a shows the results for the unilateral link creation case, while Sup. Fig. 11b shows the results for the bilateral link creation case. In both Sup. Fig. 11a and Sup. Fig. 11b, when we have only global rewiring or both under coevolution, average path length grows at a rate that is lower than $\log n$. When we have only local rewiring, the average path length grow at a rate higher than $\log n$. Parameters: $u = 0.1$, $w = 0.5$, $\beta = 0.1$, $p = 0.1$, $c = 0.2$, $d = 2$ and $l = 1$, each averaged over at least 5 runs.

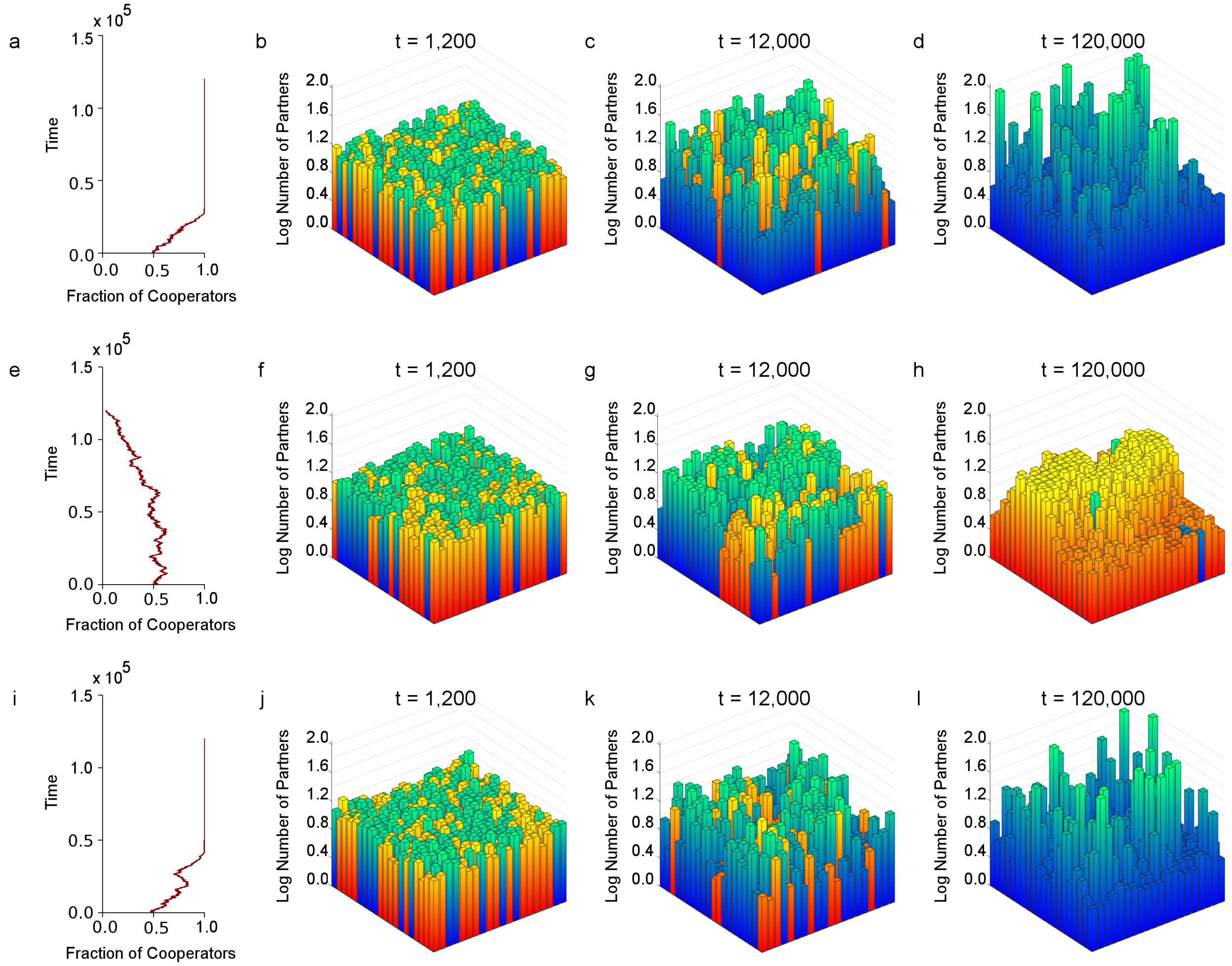

Supplementary Figure 12. **Bilateral link creation comparison of global rewiring, local rewiring and both under coevolution.** Figure shows the same comparison done in Figure 2 in the main text, but with the bilateral link creation instead. The results are the same as those for the case of unilateral link creation. Parameters: $n = 400$, $t = 1.2 \times 10^5$, $\beta = 0.1$, $u = 0.1$, $w = 0.5$, $p = 0.1$, $c = 0.2$, $d = 2$ and $l = 1$.

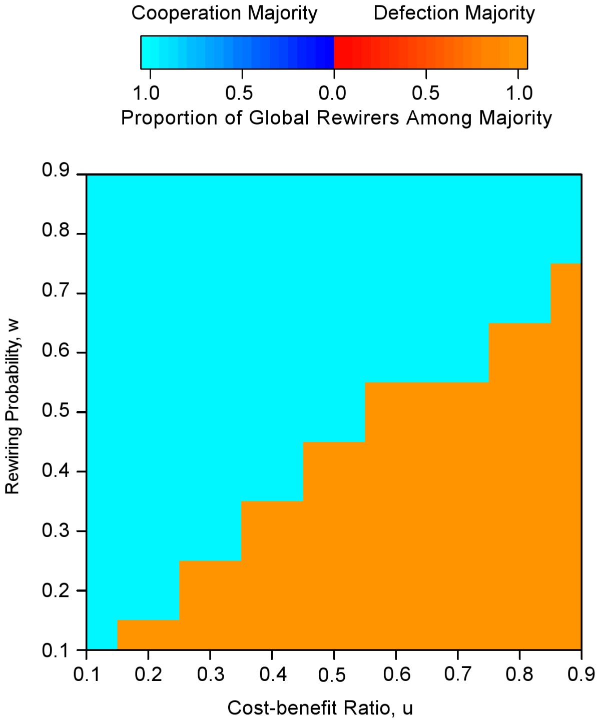

Supplementary Figure 13. **Coevolution of cooperation and long range partner rewiring preferences under bilateral link creation.** Figure shows the most common traits across the parameter space (u, w). This is same as the comparison done in Figure 3 in the main text, but for the case of bilateral link creation instead. Global cooperators are most favoured under fast partner switching w and small cost-benefit ratio u. Whereas global defectors are dominant for low w and high u. Like for Figure 3, there is a region in the parameter space where coevolutionary dynamics transits from most favouring global cooperators to global defectors. However, local cooperators can no longer be favoured. Parameters: $n = 2500$, $t = 1.5 \times 10^6$, $\beta = 0.1$, $p = 0.1$, $c = 0.2$, $d = 2$ and $l = 1$, end results averaged over an additional 3×10^4 time steps for each run, total 50 runs.

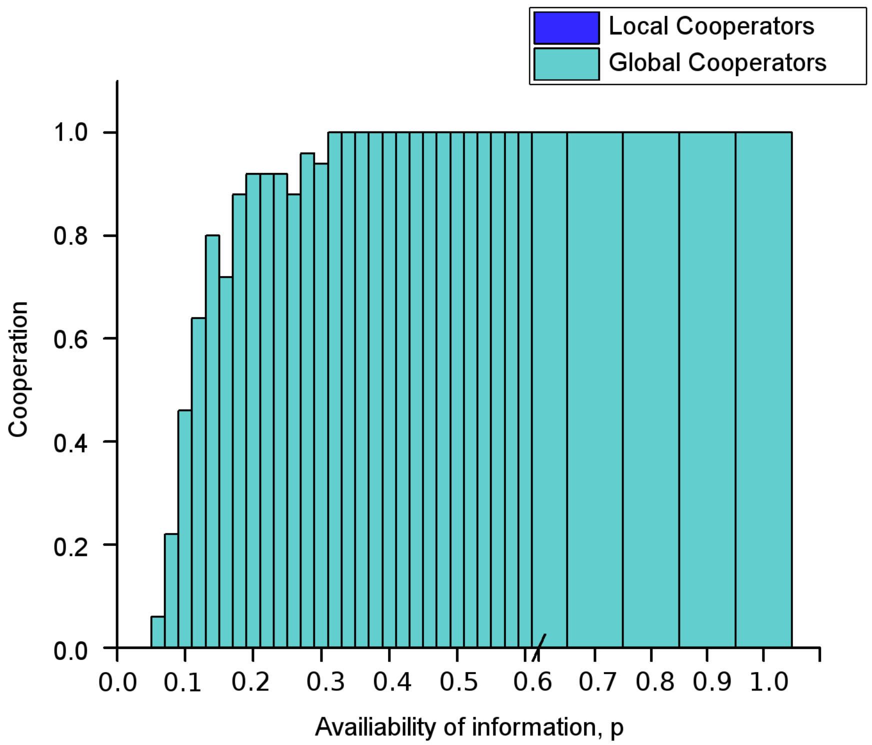

Supplementary Figure 14. **Availability of reputation information and the evolution of global cooperation under bilateral link creation.** Figure shows the proportion of local and global cooperators, at equilibrium, as a function of availability of information, p, in the case of the bilateral tie creation model. In general, the proportion of global cooperators increase with p, while local cooperators are never favoured. Parameters: $n = 2500$, $t = 1.5 \times 10^6$, $u = 0.2$, $w = 0.2$, $\beta = 0.1$, $c = 0.2$, $d = 2$ and $l = 1$, end results averaged over an additional 3×10^4 time steps for each run, total 50 runs.
